# Isoegomaketone exhibits potential as a new *Mycobacterium abscessus* inhibitor

**DOI:** 10.3389/fmicb.2024.1344914

**Published:** 2024-02-23

**Authors:** Ho Won Kim, Ji Won Lee, A-Reum Yu, Hoe Sun Yoon, Minji Kang, Byung Soo Lee, Hwan-Woo Park, Sung Ki Lee, Jake Whang, Jong-Seok Kim

**Affiliations:** ^1^Myunggok Medical Research Institute, College of Medicine, Konyang University, Daejeon, Republic of Korea; ^2^Korea Mycobacterium Resource Center (KMRC), Department of Research and Development, The Korean Institute of Tuberculosis, Osong, Republic of Korea; ^3^Department of Obstetrics and Gynecology, Konyang University Hospital, Daejeon, Republic of Korea; ^4^Department of Cell Biology, Konyang University College of Medicine, Daejeon, Republic of Korea

**Keywords:** isoegomaketone, *Mycobacterium abscessus*, minimum inhibitory concentration, biofilm formation, intracellular bactericidal activity

## Abstract

Although the incidence of *Mycobacterium abscessus* infection has recently increased significantly, treatment is difficult because this bacterium is resistant to most anti-tuberculosis drugs. In particular, *M. abscessus* is often resistant to available macrolide antibiotics, so therapeutic options are extremely limited. Hence, there is a pressing demand to create effective drugs or therapeutic regimens for *M. abscessus* infections. The aim of the investigation was to assess the capability of isoegomaketone (iEMK) as a therapeutic option for treating *M. abscessus* infections. We determined the minimum inhibitory concentration (MIC) and minimum bactericidal concentration (MBC) of iEMK for both reference and clinically isolated *M. abscessus* strains. In addition to time-kill and biofilm formation assays, we evaluated iEMK’s capability to inhibit *M. abscessus* growth in macrophages using an intracellular colony counting assay. iEMK inhibited the growth of reference and clinically isolated *M. abscessus* strains in macrophages and demonstrated effectiveness at lower concentrations against macrophage-infected *M. abscessus* than when used to treat the bacteria directly. Importantly, iEMK also exhibited anti-biofilm properties and the potential to mitigate macrolide-inducible resistance, underscoring its promise as a standalone or adjunctive therapeutic agent. Overall, our results suggest that further development of iEMK as a clinical drug candidate is promising for inhibiting *M. abscessus* growth, especially considering its dual action against both planktonic bacteria and biofilms.

## 1 Introduction

Nontuberculous mycobacteria (NTM) are opportunistic pathogens that widely exist in the environment, including in soil, natural water, and drinking water (Falkinham, 2015; Johansen et al., 2020). Human lung infections caused by NTM have seen a rapid global increase in recent decades, with a dramatic surge observed worldwide in the past few years (Koh et al., 2013; Cowman et al., 2019). Among NTM species, *Mycobacterium abscessus* (Mabs) and *Mycobacterium avium* complex (MAC) are the most commonly recognized species associated with clinically significant pulmonary diseases; these species account for over 90% of the reported cases (Wu et al., 2018; Griffith, 2019). Mabs is notorious for causing chronic pulmonary infections among susceptible hosts, such as those with cystic fibrosis (*CF*), bronchiectasis, or chronic obstructive pulmonary disease (COPD); these infections are frequently difficult to cure and associated with rapid lung function decline (Nishiuchi et al., 2017; Johansen et al., 2020). Mabs is renowned for its antibiotic resistance, stemming from both natural and acquired multidrug resistance profiles (Griffith, 2011; Nessar et al., 2012). Mabs exhibits intrinsic resistance to classical anti-tuberculosis medications and additionally to the majority of existing antimicrobials (Lee et al., 2017). Moreover, biofilms are believed to be integral in Mabs infections, with the presence of biofilm-like microcolony formation observed in patient lungs (Qvist et al., 2015; Fennelly et al., 2016). These biofilms function as robust barriers and promote tolerance in harsh environments (Mu et al., 2014), thereby complicating the treatment of Mabs infections. This situation underscores the constrained accessibility and efficacy of the few existing therapeutic options, highlighting the urgent demand for novel or redeployed drugs capable of overcoming these challenges. In particular, the critical need to identify therapeutic agents with effective anti-biofilm properties becomes evident as a pivotal strategy in addressing this clinical challenge. Hence, the search for new drug candidates that can effectively treat Mabs infections, particularly those that can disrupt biofilm formations and penetrate these protective barriers, is of paramount importance.

Recent investigations reveal the potent antimicrobial properties of *Perilla frutescens*, significantly impeding the growth of pathogenic food-borne strains (Erhunmwunsee et al., 2022; Cai et al., 2023). Perilla, commonly cultivated and utilized throughout Asia, especially in Korea, China, and Japan, plays a significant role in traditional Chinese medicine. Perilla is employed for treating various conditions, such as bronchial diseases, indigestion, and insomnia (Hou et al., 2022). Key functional components found in perilla include rosmarinic acid, perillaldehyde, luteolin, apigenin, egomaketone, and isoegomaketone (iEMK) (Zhou et al., 2014; Jin et al., 2016). There are, however, no reports concerning the antibacterial activity of *Perilla frutescens* or iEMK against Mabs. We discovered that iEMK, when used alone, exhibited antibacterial and anti-biofilm properties against Mabs, suggesting its potential as a standalone antibiotic or adjunctive therapeutic agent.

In this study, we evaluated the susceptibility of reference and clinically isolated strains of Mabs to iEMK, effectiveness against macrolide-inducible resistance, examined its capacity to disrupt biofilm formations, investigated its inhibitory effects on Mabs within macrophages, and assessed its cytotoxic impact. This represents the first comprehensive study of iEMK’s multifaceted action against Mabs, positioning it as an innovative antibiotic candidate.

## 2 Materials and methods

### 2.1 Bacterial strains and culture conditions

The *M. abscessus* reference strain (ATCC 19977) was purchased from the American Type Culture Collection. Clinical isolates were purchased from the Korea Mycobacterium Resource Center (KMRC, Osong, Korea). All *M. abscessus* strains were grown at 37°C either in liquid or on solid media. Middlebrook 7H9 broth was used as the liquid medium, which contained 0.2% glycerol (v/v) and 10% albumin-dextrose-catalase (ADC) (v/v), while the solid medium was Middlebrook 7H10 agar that contained 0.5% glycerol (v/v) and 10% oleic acid-ADC (OADC) (v/v). Stock vials of each *M. abscessus* were preserved in a deep freezer at −80°C in 7H9 broth containing 40% glycerol (v/v), 0.05% Tween 80 (v/v), and 10% OADC (v/v).

### 2.2 Antimicrobial susceptibility testing

Amikacin, ciprofloxacin, clarithromycin, doxycycline, cefoxitin, imipenem, linezolid, moxifloxacin, trimethoprim, sulfamethoxazole, and tobramycin were purchased from Sigma–Aldrich Co. (St Louis, MO, United States), and isoegomaketone was purchased from AA BLOCKS, INC (San Diego, United States). Minimum inhibitory concentrations (MICs) were determined according to CLSI guidelines, except for the composition of the medium and the concentration of isoegomaketone. To prepare the test MIC 96-well plate, drugs were mixed with 50 μL of 7H9 medium containing 10% OADC supplement in each well. The final concentrations of the tested drugs were as follows: amikacin (2 to 128 μg/mL), ciprofloxacin (0.25 to 16 μg/mL), clarithromycin (0.5 to 32 μg/mL), doxycycline (0.25 to 16 μg/mL), cefoxitin (4 to 256 μg/mL), imipenem (1 to 64 μg/mL), linezolid (1 to 64 μg/mL), moxifloxacin (0.125 to 8 μg/mL), trimethoprim/sulfamethoxazole (0.5/9.5 to 32/608 μg/mL), and tobramycin (1 to 8 μg/mL). For MIC determination, all Mabs strains were inoculated in 7H9 medium and incubated at 37°C for 3 to 5 days. Cultures were homogenized with 7H9 media and then adjusted to a McFarland of 0.5 with sterile saline. Then, the cells were diluted 100-fold for inoculation in 2X cation-adjusted Mueller-Hinton broth (CAMHB) containing 50 μg/mL 2,3-diphenyl-5-thienyl-(2)-tetrazolium chloride (STC; TCI, Tokyo, Japan) for mycobacterial staining, and 50 μL was inoculated into each well of the prepared MIC test 96-well plate. For amikacin, ciprofloxacin, doxycycline, cefoxitin, imipenem, linezolid, moxifloxacin, trimethoprim/sulfamethoxazole, and tobramycin, the MICs were determined after 3, 5, and 7 days of incubation. In addition, the plates were submitted to an extended incubation for clarithromycin MIC testing, which allowed the evaluation of macrolide-inducible resistance with readings taken after 3, 5, 7, 10, and 14 days of incubation at 30°C. The MIC was defined as the lowest concentration at which no visual bacterial growth occurred, indicating complete inhibition of bacterial growth. Susceptibility was assessed following the guidelines set by CLSI for breakpoints (Supplementary Table S1). The MIC of isoegomaketone was determined using the same method, with the final isoegomaketone test concentrations ranging from 1 to 1,024 μg/mL. MICs were determined after 3-and 5-day incubations. All assays were performed in triplicate.

### 2.3 Bactericidal/static activity determination

Minimum bactericidal concentrations (MBCs) of isoegomaketone were determined for all *M. abscessus* strains to differentiate between bacteriostatic and bactericidal activities. After 4 days of incubation with isoegomaketone at different concentrations, the wells with drug concentrations that exceeded the MIC were resuspended. Then, 100 μL of medium from each well was serially diluted (by 10-fold) and inoculated into a 7H10 plate. The plates were incubated at 37°C for 5 days, then, the number of colonies was counted. The MBC value was the lowest drug concentration at which no colonies grew. Antibiotics were classified as bactericidal if the MBC/MIC ratio was ≤4 and as bacteriostatic if the ratio exceeded 4 (Levison, 2004; Benjamin et al., 2012).

### 2.4 Time–kill assays

The time-kill assays were performed by adding isoegomaketone, clarithromycin, or amikacin at 1X MIC antibiotic concentrations and the inoculum (density 1 × 10^5^ CFU/mL) into 1 mL of 7H9 broth with 10% OADC. As a control, aliquots containing sterile distilled water instead of antibiotics were included. All tubes were incubated at 37°C for 5 days. The bacteria were enumerated at 24 h intervals by plating serial dilutions on 7H10 agar plates. CFUs were counted after an additional 4 days of incubation at 37°C. A CFU count curve was drawn over time to characterize the effect of the antibiotics at different concentrations.

### 2.5 Crystal violet assay of biofilms

For biofilm formation, 500,000 CFUs of the reference and clinically isolated *M. abscessus* strains were inoculated in 7H9 medium containing 10% OADC in U-bottom 96-well plates. Isoegomaketone at final concentrations of 4, 16, or 64 μg/mL was then added to the wells in quintuplicate. Control wells received an equal volume of DMSO and were incubated at 37°C for approximately 10 days without shaking. After the formation of the bacterial biofilm, the medium was removed from wells by pipetting underneath the biofilm at the interface. Biofilms were dried in a biosafety cabinet; then, the biofilms were incubated with 200 μL of 1% crystal violet for 30 min. The stain was removed, and the biofilm was gently washed three times with PBS. The bound crystal violet was then extracted by a 10 min incubation at 37°C with 200 μL of 95% ethanol. The absorbance of the extracted crystal violet was measured at 570 nm on a spectrophotometer.

### 2.6 Cell viability assay

The viability of BMDMs and THP-1 cells treated with isoegomaketone was evaluated using a CCK-8 assay kit (Dojindo, Japan). BMDMs were obtained by flushing the femurs and tibias of eight-week-old female C57BL/6 J mice purchased from DBL (Gyeonggi-do, Pyeongtaek-si, Korea) with DMEM (Welgene, Gyeongsan-si, Gyeongsangbuk-do, Korea). Bone marrow cells were differentiated into BMDMs by exposure to 10 ng/mL recombinant mouse macrophage colony-stimulating factor (M-CSF; PeproTech) at 37°C in a 5% CO2 incubator for 6 days. THP-1 cells were obtained from the American Type Tissue Collection (ATCC) and were maintained in DMEM with 10% FBS. THP-1 differentiation was induced by adding 100 nM phorbol 12-myristate 13-acetate (PMA; Sigma) and incubating at 37°C in a 5% CO2 incubator overnight. BMDMs and differentiated THP-1 cells were seeded in a 96-well plate at a density of 1 × 10^5^ cells per well. Isoegomaketone was added to the cells at various concentrations, and the samples were incubated at 37°C for 48 h. For the cell viability assay, 20 μL of CCK-8 reagent (10% media volume) was added to each well, and the incubation was continued for 1.5 h. The resulting color was assessed at 450 nm. A SpectraMax^®^ M3 Multi-Mode Microplate Reader (Molecular Devices, Sunnyvale, CA, USA) was used for each assay.

### 2.7 Intracellular killing and concentration-killing assays

To assess the activity of isoegomaketone against intracellular *M. abscessus*, BMDMs and differentiated THP-1 cells (1.5 × 105 cells/well) were dispensed into 48-well plates (SPL, New York, NY, USA) and then infected with the reference or clinically isolated *M. abscessus* strains at a multiplicity of infection (MOI) of 1:1. After 4 h of infection, the cells were washed with PBS to remove the uninfected bacteria and incubated with DMEM containing 20 μg/mL amikacin for 1.5 h to kill the remaining extracellular *M. abscessus*. The cells were then washed with PBS and a broth medium with various concentrations of isoegomaketone was added. After 24 h or 48 h of incubation at 37°C and 5% CO2, BMDMs and differentiated THP-1 cells were extensively washed with PBS, lysed with 0.05% Triton X-100, and then serially diluted with PBS. The number of CFUs was quantified by plating serial dilutions of lysates on M7H10 agar plates.

### 2.8 Ethics statement

The animals were housed in an SPF barrier room under controlled conditions on a 12 h light–dark cycle and a constant temperature (25°C). The experiments were performed in accordance with the Animal Care and the Guiding Principles for Animal Experiments Using Animals were approved by the University of Konyang Animal Care and Use Committee (21-07-E-01).

### 2.9 Statistical analysis

All experiments were performed in triplicate. Statistical analyses were performed using GraphPad Prism 6.0 software. For the biofilm formation assay, cell viability assay, and intracellular bactericidal assay, one-way ANOVA was employed to identify significant differences between the groups. In the case of time–kill assays, a two-way ANOVA was utilized to determine significant differences between the groups. Error bars represent the standard deviation (SD). Differences were considered statistically significant at a *p* value of <0.05.

## 3 Results

### 3.1 Antimicrobial susceptibility and bactericidal/bacteriostatic activities of iEMK against reference and clinically isolated Mabs strains

A total of 10 Mabs strains, including 2 reference strains and 8 clinically isolated strains, were studied. Morphological observation indicated that 5 out of the 8 Mabs isolates had smooth morphotypes, while 3 exhibited rough morphotypes. The minimum inhibitory concentration (MIC), minimum bactericidal concentration (MBC), and MBC/MIC ratio of iEMK in the reference and clinically isolated strains are shown in Table 1. iEMK showed effective activity against most Mabs strains. The two reference and two clinical strains had MICs of 128 μg/mL, and the 6 other clinical strains had MICs of ≤64 μg/mL. Additionally, there was no difference in MIC based on morphological differences. A total of 10 Mabs strains had MBCs of 128 to 256 μg/mL, and the MBC/MIC ratio ranged from 2 to 4. Since iEMK had MBC/MIC ratios ≤4 against the strains, the bactericidal effect was evaluated in these strains. The results of antimicrobial susceptibility testing, in accordance with the recommendations from The Clinical & Laboratory Standards Institute (CLSI) 2011, for both the reference and clinically isolated strains are shown in Table 2. All of the strains were susceptible or intermediate susceptible to amikacin, cefoxitin, imipenem, linezolid, and tigecycline. Eight of the strains were resistant to moxifloxacin, and six were resistant to ciprofloxacin and tobramycin. Furthermore, all strains showed resistance to doxycycline and trimethoprim/sulfamethoxazole. All nine strains were susceptible to clarithromycin, except for a single clinically isolated strain (KMRC 00136–61,203) that showed resistance. Therefore, iEMK is an effective drug candidate for clarithromycin, doxycycline, and trimethoprim/sulfamethoxazole resistant isolates.

**Table 1:** The MIC, MBC, and antibacterial activity of isoegomaketone in the reference and clinically isolated *M. abscessus* strains.

		Morphotype	MIC	MBC	MBC/MIC Ratio	Antibacterial Activity
Reference strains	ATCC 19977	Rough	128	256	2	Bactericidal
	ATCC 19977	Smooth	128	256	2	Bactericidal
Clinical strains	KMRC 00136–61,040	Rough	128	256	2	Bactericidal
	KMRC 00136–61,041	Smooth	64	128	2	Bactericidal
	KMRC 00136–61,198	Smooth	64	128	2	Bactericidal
	KMRC 00136–61,199	Smooth	64	128	2	Bactericidal
	KMRC 00136–61,202	Rough	128	256	2	Bactericidal
	KMRC 00136–61,203	Smooth	32	128	4	Bactericidal
	KMRC 00136–61,308	Rough	32	128	4	Bactericidal
	KMRC 00136–61,309	Smooth	32	128	4	Bactericidal

**Table 2:** MICs of antibiotics in the reference and clinically isolated *M. abscessus* strains.

		Mnimum inhibitory concentration (MIC) (μg/ml)									
		AMI^a^	CIP^a^	CLR^a^	DOX^a^	FOX^a^	IMI^a^	LZD^a^	MXF^a^	SXT^a^	TOB^a^
Reference strains	ATCC 19977 Rough	4	4	1^b^	≥16	8	8	4	4	≥32/608	4
	ATCC 19977 Smooth	≤2	2	1^2^	≥16	32	2	4	4	16/304	4
Clinical strains	KMRC 00136–61,040	8	16	≤0.5^2^	≥16	64	16	8	4	≥32/608	8
	KMRC 00136–61,041	≤2	2	≤0.5^2^	≥16	32	2	2	2	4/76	4
	KMRC 00136–61,198	4	4	≤0.5	16	32	2	2	4	8/152	8
	KMRC 00136–61,199	4	8	≤0.5	≥16	32	2	8	≥8	16/304	8
	KMRC 00136–61,202	16	4	≤0.5	≥16	32	8	4	≥8	≥32/608	≥8
	KMRC 00136–61,203	≤2	8	≥32	≥16	32	2	8	8	4/76	8
	KMRC 00136–61,308	4	2	1^2^	≥16	32	2	4	4	≥32/608	2
	KMRC 00136–61,309	4	2	≤0.5	≥16	32	2	2	2	16/304	8

AMI, Amikacin; CIP, Ciprofloxacin; CLR, Clarithromycin; DOX, Doxycycline; FOX, Cefoxitin; IMI, Imipenem; LZD, Linezolid; MXF, Moxifloxacin; SXT, Trimethoprim/Sulfamethoxazole; TOB, Tobramycin.

a These breakpoints are recommended by the Clinical and Laboratory Standards Institute (CLSI 2011) for rapidly growing mycobacteria.

b inducible resistance, According to the CLSI guidelines, macrolide inducible resistance is defined as an increase in the MIC of clarithromycin from ≤ 2 μg/mL at day 5 to ≥ 8 μg/mL at day 14 of incubation.

### 3.2 *In vitro* antibacterial activity of iEMK against Mabs

**Figure 1:**
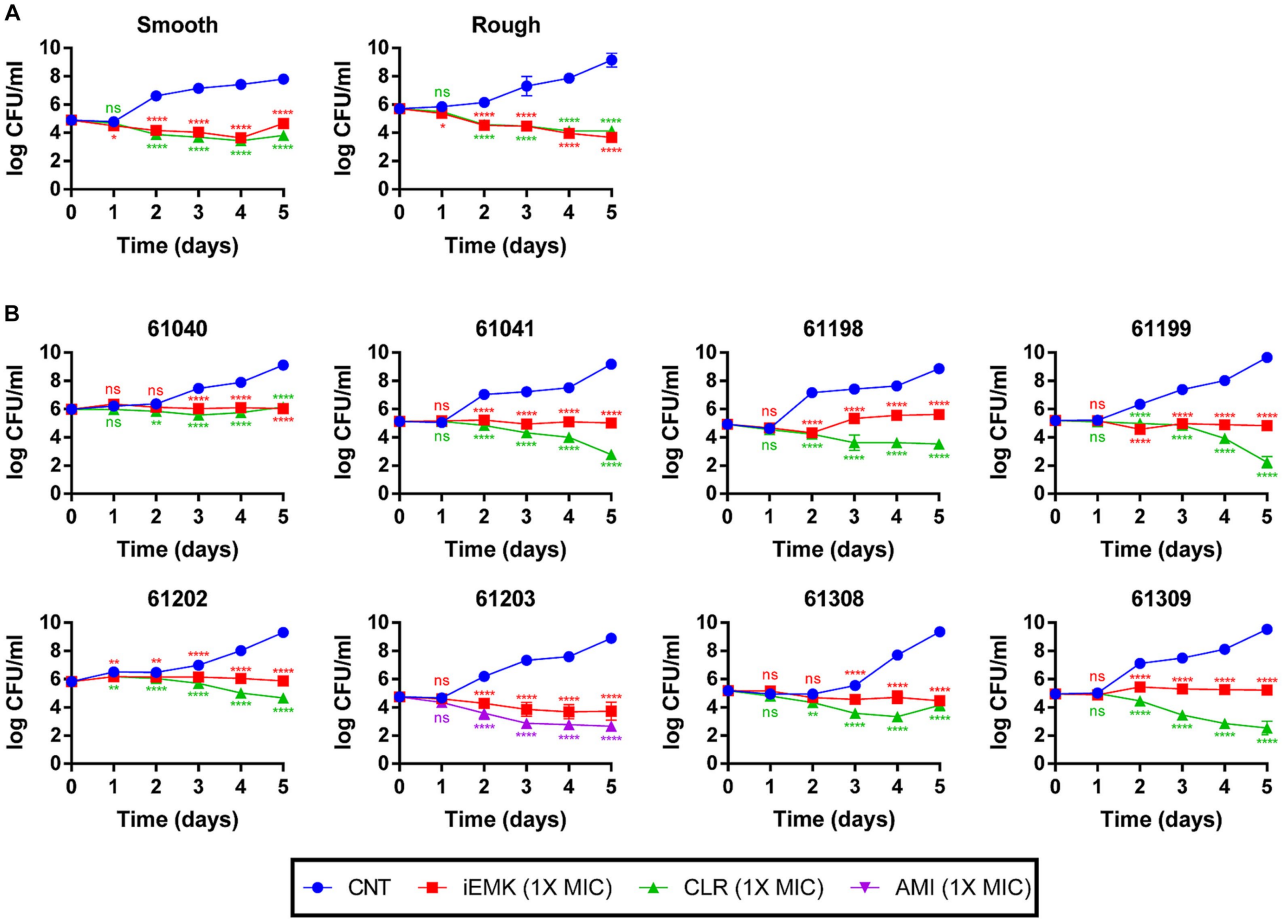
Time-Kill kinetics of isoegomaketone against reference and clinically isolated strains of *M. abscessus*. Time-kill curves over 5 days compare the negative control, isoegomaketone, and either clarithromycin or amikacin against both reference and clinically isolated *M. abscessus* strains. Each panel represents a different strain, with **(A)** showing the reference strain and **(B)** the clinical isolates. Curves are depicted for the negative control (CNT, blue circles), isoegomaketone at 1x MIC (iEMK, red squares), clarithromycin at 1x MIC (CLR, green triangles), and amikacin at 1x MIC (AMI, purple triangles). Clarithromycin and amikacin were used as positive controls. Data are presented as the mean ± standard deviation (SD) from triplicates at each concentration. Significance levels: ^*^p < 0.05; ^**^p < 0.01; ^****^p < 0.0001; ns = not significant.

In addition to the MIC results, *in vitro* time-kill assays were conducted using iEMK and the first-line drug clarithromycin or amikacin (Figure 1). In general, time–kill curves for the Mabs reference strains showed a greater decrease in bacterial population size after 2 to 4 days than what was observed for the clinically isolated strains when exposed to 1 X MIC iEMK. All Mabs strains exhibited a slow decrease in population size in response to treatment with positive controls, such as clarithromycin or amikacin, over a 5-day period. However, when exposed to iEMK, the reference Mabs strains exhibited only a slight decrease in population size, similar to what was seen with the positive control treatment, whereas in clinically isolated strains, iEMK had bacteriostatic activity.

### 3.3 iEMK against macrolide-inducible resistance in Mabs

**Figure 2:**
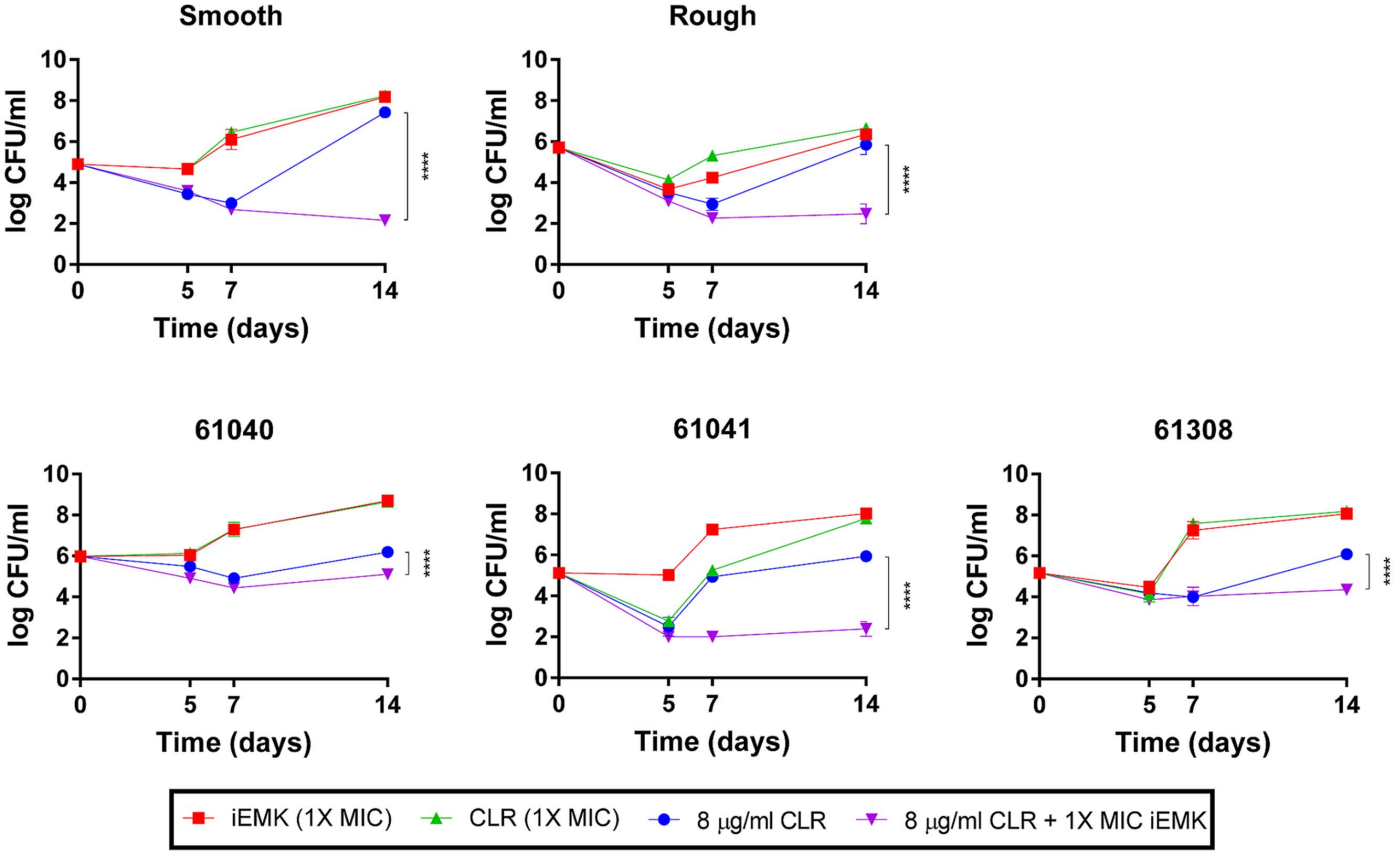
Isoegomaketone effect on macrolide-inducible resistance in *M. abscessus* strains. This figure displays the time-kill kinetics of isoegomaketone (iEMK) on *M. abscessus* strains with macrolide-inducible resistance, as outlined by CLSI guidelines. Macrolide-inducible resistance is demonstrated by an increase in clarithromycin (CLR) MIC from ≤2 μg/mL on day 5 to ≥8 μg/mL by day 14 of incubation. Time-kill curves were plotted for (green) CLR at 1X MIC, (red) iEMK at 1X MIC, (blue) CLR at 8 μg/mL, and (purple) a combination of 8 μg/mL CLR and 1X MIC iEMK against *M. abscessus* strains. Data are presented as the mean ± standard deviation (SD) from triplicates at each concentration. Significance is marked by ^****^p < 0.0001. iEMK, isoegomaketone; CLR, clarithromycin.

The phenomenon of macrolide-inducible resistance poses a formidable challenge in the clinical management of Mabs infections. This resistance significantly undermines the efficacy of standard macrolide therapy, which is essential for treating these infections (Nash et al., 2009; Brown-Elliott et al., 2012). Given the critical nature of this issue, our study aimed to elucidate the potential of iEMK in addressing macrolide-inducible resistance. In our extended 14-day *in vitro* time-kill assays, we assessed the effects of combining iEMK with CLR on Mabs strains exhibiting this type of resistance. Our results, depicted in Figure 2, demonstrate that CLR at 8 μg/mL initially suppressed bacterial growth in strains with macrolide-inducible resistance. Notably, by day 14, these strains began to exhibit regrowth. In contrast, the combination of iEMK with CLR prevented such regrowth, effectively showcasing iEMK’s ability to inhibit the development of macrolide-inducible resistance.

### 3.4 iEMK inhibits Mabs biofilm formation

**Figure 3:**
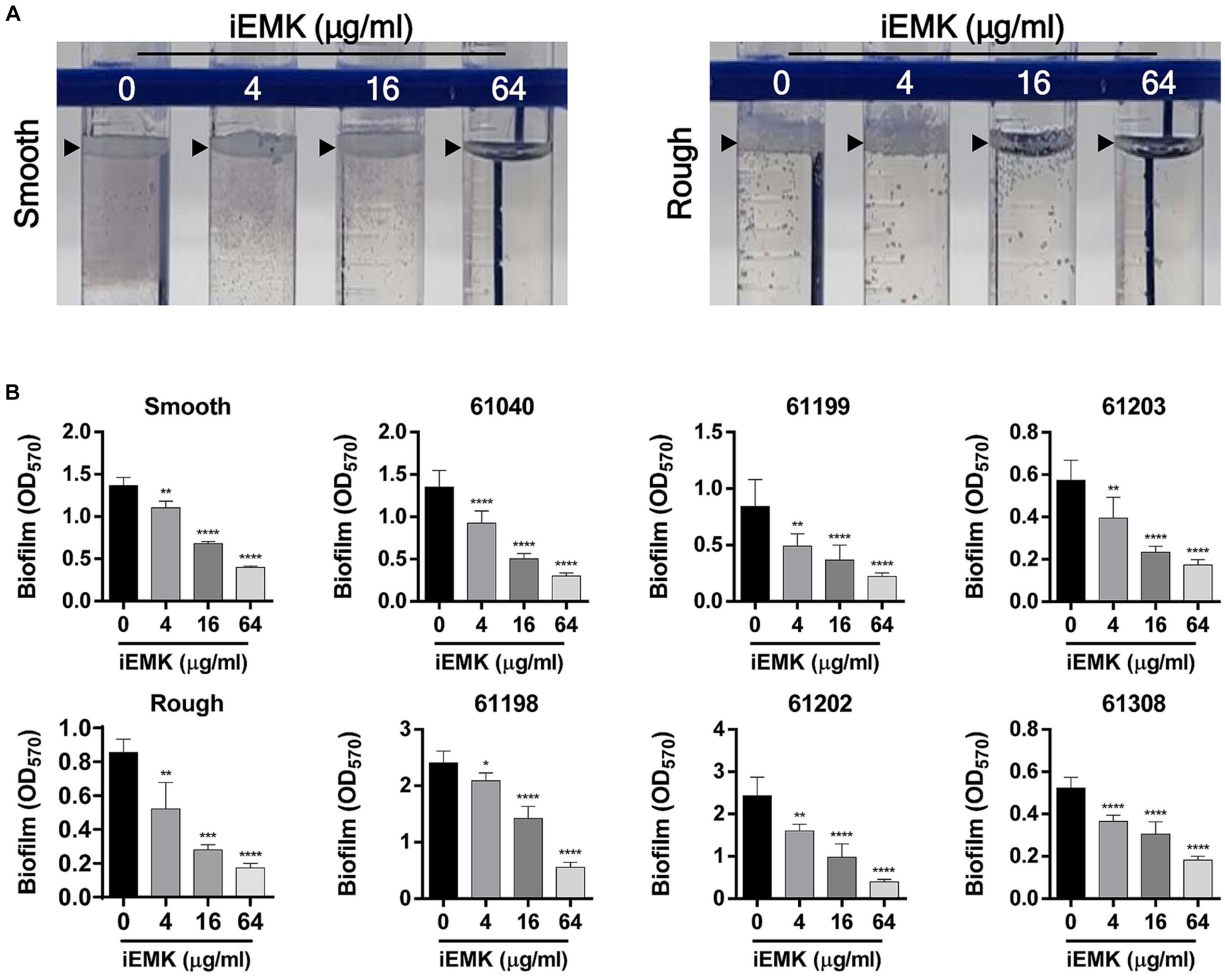
Effect of isoegomaketone on biofilm formation in reference and clinically isolated strains of *M. abscessus*. Isoegomaketone (iEMK) was applied at varying concentrations to both reference and clinically isolated strains of *M. abscessus* to assess its biofilm inhibitory effect over 7 days at 37°C. **(A)** Represents biofilm development at the liquid-air interface in reference strains, displaying the characteristic biofilm architecture. **(B)** Quantifies biofilm biomass using crystal violet staining, with absorbance measured spectrophotometrically at 570 nm post ethanol extraction. The dose–response graphs depict a reduction in biofilm mass correlating with increased iEMK concentrations. Error bars represent standard deviation from triplicate measurements. Significance denoted as ^*^p < 0.05; ^**^p < 0.01; ^****^p < 0.0001; ns indicates not significant.

Biofilms are thought to play an essential role in Mabs infections, and there is evidence of microcolony formations in patient lungs that resemble biofilms (Qvist et al., 2015; Fennelly et al., 2016). Moreover, *in vitro* biofilm models of Mabs have reduced susceptibility to several first-line antibiotics, including cefoxitin, amikacin, and clarithromycin (Greendyke and Byrd, 2008; Marrakchi et al., 2014). Thus, we examined the antibiofilm effects of iEMK. Eight of the reference and clinically isolated Mabs strains had average OD570 values ranging from 0.52 to 2.43 (Figure 3). The remaining clinically isolated strains (61,041 and 61,309) had OD570 values below 0.20 and were considered non-biofilm-forming isolates. Our investigation into iEMK’s antibiofilm activity demonstrated a dose-dependent reduction in biofilm formation across the tested strains when treated with concentrations of 4, 16, or 64 μg/mL (Figure 3). This observation was particularly notable as iEMK effectively inhibited biofilm formation at significantly reduced concentrations, showcasing its potential as an effective antibiofilm agent against both reference and clinically isolated Mabs strains, including the notably resistant 61,040 strain (Figure 3).

### 3.5 Cell viability assays of iEMK using murine bone marrow-derived macrophages and human monocytic leukemia cells

**Figure 4:**
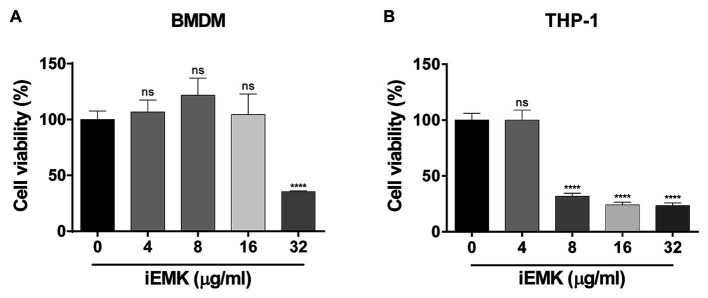
The effect of isoegomaketone on BMDMs **(A)** and THP-1 **(B)** cell viability. The effect of iEMK on BMDMs and THP-1 cell viability was evaluated 48 h after treatment with different concentrations of iEMK. All data are shown as the mean ± SD (*n* = 3). The results shown are from one representative experiment, ^****^p < 0.0001; ns, not significant.

To determine whether iEMK induces cell death, cell viability at different concentrations of iEMK was assessed 48 h after treatment using the CCK assay. The results showing the effects of iEMK on the viability of murine bone marrow-derived macrophages (BMDMs) and differentiated human monocytic cells (THP-1) are presented in Figure 4. When the concentration of iEMK was 16 μg/mL, the survival rates of the BMDMs were nearly 100% after 48 h of exposure. In contrast, the differentiated THP-1 cells exhibited reduced cell viability compared to the control group when exposed to iEMK concentrations higher than 8 μg/mL.

### 3.6 Intracellular bactericidal activity of iEMK against Mabs in murine bone marrow-derived macrophages and human macrophages

**Figure 5:**
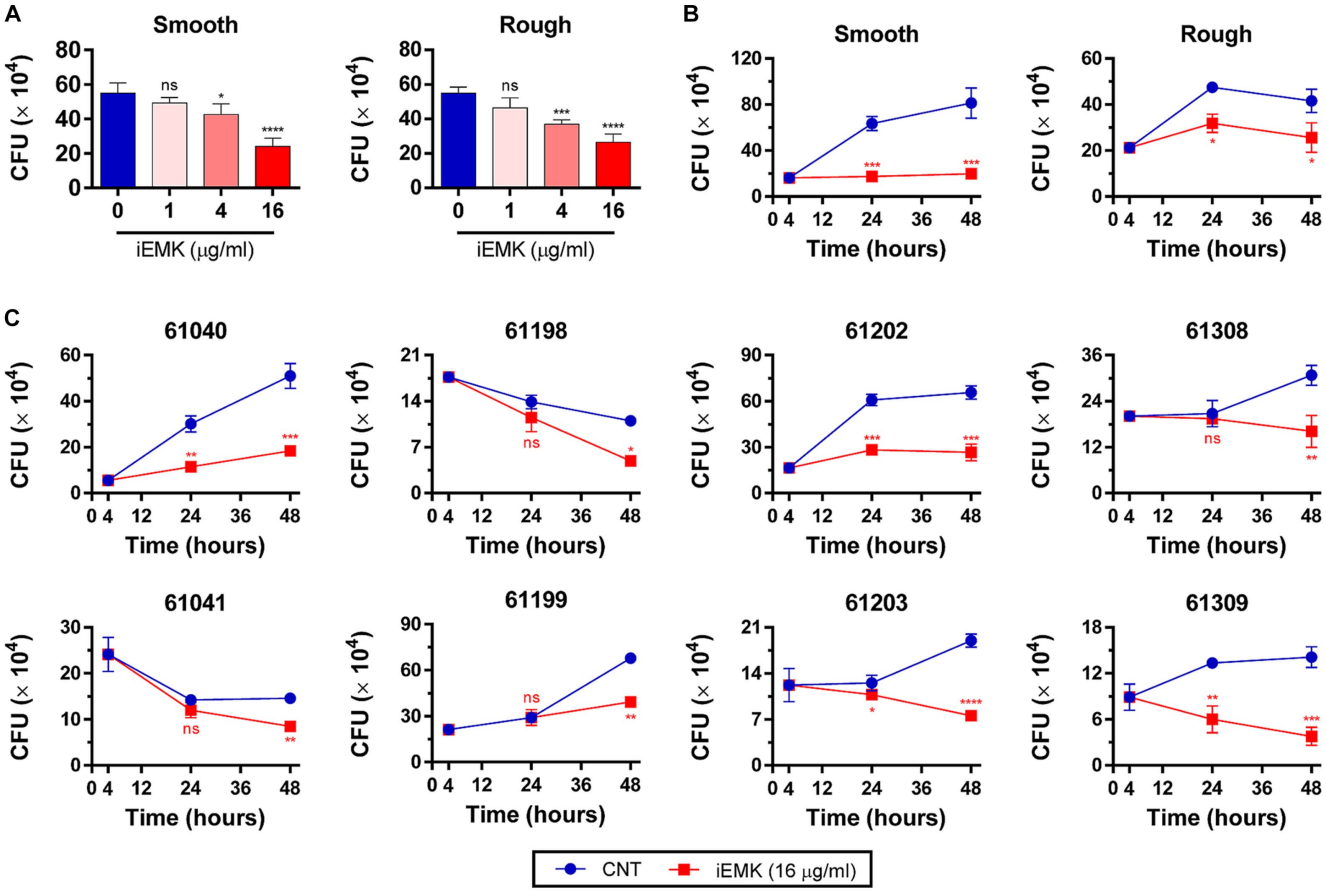
Isoegomaketone suppresses intracellular growth of reference and clinically isolated Strains of *M. abscessus* in BMDMs. Intracellular CFU analyses were performed on BMDMs infected with the reference and clinically isolated strains of *M. abscessus* (MOI = 1:1). Initial CFU counts determined at 4 h post-infection set the baseline for infection levels. **(A)** Reference strains were treated with varying concentrations of isoegomaketone (iEMK) and analyzed 48 h post-treatment. **(B)** Reference and **(C)** clinically isolated strains were treated with 16 μg/mL iEMK, with effects assessed at 24 and 48 h post-treatment. Error bars indicate the standard deviation from triplicate experiments. Statistical significance is indicated as ^*^p < 0.05; ^**^p < 0.01; ^***^p < 0.001; ****p < 0.0001; ns denotes a non-significant difference.

**Figure 6:**
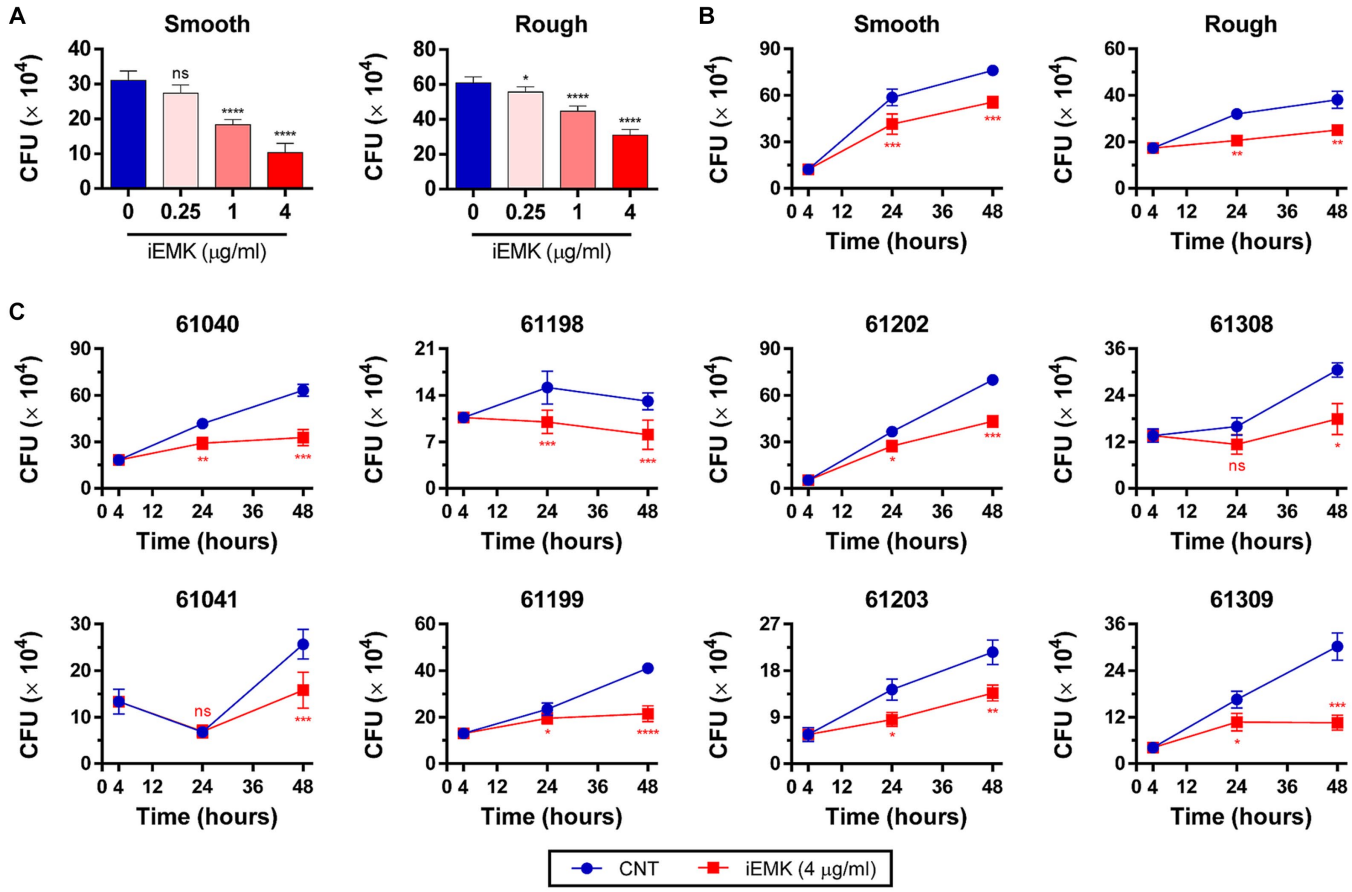
Isoegomaketone suppresses intracellular growth of reference and clinically isolated strains of *M. abscessus* in THP-1 Cells. Intracellular CFU analyses were performed on PMA-differentiated THP-1 cells infected with the reference and clinically isolated strains of *M. abscessus* (MOI = 1:1). Initial CFU counts determined at 4 h post-infection set the baseline for infection levels. **(A)** Reference strains were treated with varying concentrations of isoegomaketone (iEMK) and analyzed 48 h post-treatment. **(B)** Reference and **(C)** clinically isolated strains were treated with 4 μg/mL iEMK, with effects assessed at 24 and 48 h post-treatment. Error bars indicate the standard deviation from triplicate experiments. Statistical significance is indicated as ^*^p < 0.05; ^**^p < 0.01; ^***^p < 0.001; ^****^p < 0.0001; ns denotes a non-significant difference.

The efficacy of a drug against Mabs in a broth medium may not always align with its effectiveness against intracellular Mabs (Rastogi et al., 1987). In addition, Mabs can invade and replicate within macrophages, which indicates its ability to evade antimicrobial defenses in macrophages (Oberley-Deegan et al., 2010; Abdalla et al., 2015). Consequently, it is important to examine iEMK inhibition of Mab growth in intracellular environments. Thus, we tested the activity of iEMK in terms of intracellular bacterial replication. We infected BMDMs and THP-1 cells with the reference and clinically isolated Mabs strains, and 4 h later, we washed the cells to remove extracellular Mabs and supplemented the media with iEMK. After treatment, we estimated intracellular Mabs growth by plating and counting CFUs at 24 and 48 h postinfection. Our analysis demonstrated a dose-dependent decrease in CFUs of reference Mabs strains treated with iEMK within BMDMs and THP-1 cells, particularly noted at 48 h postinfection (Figures 5A, 6A). Moreover, iEMK showed significant growth inhibition for both reference and clinically isolated Mabs strains at 24 and 48 h post-treatment. Specifically, growth inhibition for both reference and clinically isolated Mabs strains was observed in BMDMs at iEMK concentrations ranging from 1/8 to 1/2 MIC (Figures 5B,C). In a similar vein, bacterial growth was significantly reduced in THP-1 cells treated with iEMK at concentrations from 1/32 to 1/8 MIC (Figures 6B,C).

## 4 Discussion

Mabs is a serious threat to human health and is naturally resistant to a broad range of antibiotics; furthermore, there has been a lack of newly discovered active molecules. To date, the treatment of Mabs infection has been particularly difficult, and there has been a low cure rate (Kwak et al., 2019; Johansen et al., 2020). Although certain antibiotics, such as AMK, CFX, and IMP, demonstrate effectiveness against Mabs, only CLR has compelling clinical efficacy for the treatment of Mabs-induced pulmonary disease (Nie et al., 2014). Nevertheless, clarithromycin-based therapy regimens are less efficient, and clarithromycin resistance in some isolates results in treatment failure (Hanh et al., 2020). Therefore, new antimicrobial agents and new targets for this organism are needed.

In this study, we found that iEMK mildly inhibited the growth of the reference and clinically isolated Mabs strains. The MIC and MBC of iEMK against Mabs were 32 ~ 128 and 128 ~ 256 μg/mL, respectively. Despite the relatively high MIC concentrations, there is still a positive outlook, as the MBC/MIC ratio of iEMK for Mabs in this study was indicative of bactericidal activity. Furthermore, we demonstrated the activity of iEMK against different Mabs morphotypes, a set of clinical isolates, and strains resistant to trimethoprim/sulfamethoxazole, doxycycline, and clarithromycin.

Clarithromycin is a crucial agent for the treatment of complex Mabs infections (Shirata et al., 2020). However, the primary reason for treatment failure is inducible resistance (Nash et al., 2009; Lee et al., 2014). Thus, there have been extensive endeavors to identify the best partner for to enhance CLR treatment efficacy. The combination of CLR with iEMK was tested in macrolide-inducible resistant Mabs strains; the combination reduced or prevented inducible resistance following 14 days of exposure.

Biofilms provide robust physical barriers that prevent the penetration of harmful compounds, block oxygen and nutrient access, and enhance Mabs tolerance to harsh conditions (Mu et al., 2014). Mabs has demonstrated its ability to thrive within biofilms in the human lung, often embedded in the alveolar walls of end-stage lung disease (Qvist et al., 2015). Moreover, Mabs is a multidrug-resistant NTM known for its biofilm-forming capabilities, and it is increasingly prevalent among patients with chronic and structural lung conditions (Ojha et al., 2005). Therefore, substances that selectively inhibit biofilm formation during antibiotic treatment represent a novel therapeutic approach for clearing Mabs (Kostakioti et al., 2013). In the present study, our findings highlight the significant potential of iEMK not just as an antimicrobial agent but also for its anti-biofilm properties. While iEMK demonstrated moderate bactericidal activity with MIC values ranging from 32 to 128 μg/mL against both reference and clinically isolated Mabs strains, its ability to effectively inhibit biofilm formation at subinhibitory concentrations (4 μg/mL, 1/32 MIC) is particularly noteworthy. Such dual functionality suggests iEMK’s utility in not only directly targeting Mabs but also in mitigating the challenges posed by biofilm-associated drug resistance. This dual approach could be crucial for developing more effective treatments against Mabs infections, underscoring the need for further research into iEMK’s mechanisms of action and its potential integration into existing treatment regimens.

Mabs is a rapidly growing NTM species that infects human macrophages in the lungs and skin (Griffith et al., 1993; Petrini, 2006). These infections lead to various clinical syndromes in humans, and similar to other pathogenic mycobacterial species, Mabs has the capacity to evade macrophage antimicrobial systems by multiplying within them (Takaki et al., 2013). Therefore, the identification of compounds that can penetrate the cell membrane to eliminate intracellular Mabs is crucial. In the present study, we found that iEMK effectively inhibited the growth of intracellular Mabs in murine bone marrow-derived macrophages (mBMDMs) and THP-1 cells. Cell viability assays indicated that BMDMs and THP-1 cells maintained high viability (>97%) when exposed to iEMK at concentrations of ≤16 μg/mL and ≤ 4 μg/mL, respectively. The highest concentration with viability was significantly lower than the MICs of the reference and clinically isolated Mabs strains for iEMK. This suggests that iEMK might enhance the antimicrobial capabilities of macrophages or accumulate within macrophages.

There are several limitations in our study. The clinical utilization of iEMK is significantly restricted by its relatively high MIC. Therefore, the development of novel applications, appropriate administration methods, or enhanced analogs of iEMK is imperative for effectively surmounting the challenges related to clinical application. Moreover, the bactericidal activity of iEMK against Mabs was solely examined *in vitro* and within macrophages, and these findings may not entirely replicate *in vivo* responses. Further research is needed to examine the efficacy of iEMK in an animal model, assess its pharmacokinetic/pharmacodynamic (PK/PD) profile, and investigate potential synergy between iEMK and other compounds for treating Mabs infection.

In conclusion, both *in vitro* and intracellular bacterial growth assays demonstrated that iEMK possesses antimicrobial activity against Mabs and the ability to inhibit biofilm development, making it a promising drug candidate for Mabs treatment.

## Data availability statement

The raw data supporting the conclusions of this article will be made available by the authors, without undue reservation.

## Ethics statement

The animal study was approved by University of Konyang Animal Care and Use Committee. The study was conducted in accordance with the local legislation and institutional requirements.

## Author contributions

HK: Conceptualization, Data curation, Investigation, Writing – original draft. JL: Conceptualization, Data curation, Investigation, Writing – original draft. A-RY: Data curation, Investigation, Writing – original draft. HY: Data curation, Investigation, Writing – original draft. MK: Data curation, Investigation, Writing – original draft. BL: Data curation, Investigation, Resources, Writing – original draft. H-WP: Conceptualization, Resources, Writing – original draft. SL: Conceptualization, Resources, Writing – original draft. JW: Conceptualization, Resources, Writing – original draft. J-SK: Conceptualization, Writing – original draft.
