# Supplementary material for: Isoegomaketone exhibits potential as a new Mycobacterium abscessus inhibitor
Source: Front Microbiol. 2024 Feb 23;15:1344914. doi: 10.3389/fmicb.2024.1344914 (PMC10996855; doi:10.3389/fmicb.2024.1344914)
Supplement: Supplementary file 1 [file Table_1.docx]

**Supplemental materials**

Supplementary Table 1. Breakpoints of each drug, according to the CLSI recommendations.

| Antibiotic | MIC (μg/ml) | | |
| --- | --- | --- | --- |
|  | Susceptible breakpoint | Intermediate susceptible breakpoint | Resistant breakpoint |
| Amikacin | ≤16 | 32 | ≥64 |
| Ciprofloxacin | ≤1 | 2 | ≥4 |
| Clarithromycin | ≤2 | 4 | ≥8 |
| Doxycycline | ≤1 | 2-4 | ≥8 |
| Cefoxitin | ≤16 | 32-64 | ≥128 |
| Imipenem | ≤4 | 8-16 | ≥32 |
| Linezolid | ≤8 | 16 | ≥32 |
| Moxifloxacin | ≤1 | 2 | ≥4 |
| Trimethoprim/Sulfamethoxazole | ≤2/38 | - | ≥4/76 |
| Tobramycin | ≤2 | 4 | ≥8 |
| CLSI, Clinical and Laboratory Standards Institute; MIC, minimum inhibitory concentration. | | | |
